# Characterizing the size and shape of sea ice floes

**DOI:** 10.1038/srep10226

**Published:** 2015-05-27

**Authors:** Marco Gherardi, Marco Cosentino Lagomarsino

**Affiliations:** 1 Università degli Studi di Milano, Dip. Fisica, Via Celoria 16, 20133 Milano, Italy; 2 I.N.F.N. Milano, Via Celoria 16, 20133 Milano, Italy; 3 CNRS, UMR 7238; 4 University Pierre et Marie Curie, 15 rue de l'École de Médecine Paris, France

## Abstract

Monitoring drift ice in the Arctic and Antarctic regions directly and by remote sensing is important for the study of climate, but a unified modeling framework is lacking. Hence, interpretation of the data, as well as the decision of what to measure, represent a challenge for different fields of science. To address this point, we analyzed, using statistical physics tools, satellite images of sea ice from four different locations in both the northern and southern hemispheres, and measured the size and the elongation of ice floes (floating pieces of ice). We find that (i) floe size follows a distribution that can be characterized with good approximation by a single length scale 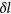, which we discuss in the framework of stochastic fragmentation models, and (ii) the deviation of their shape from circularity is reproduced with remarkable precision by a geometric model of coalescence by freezing, based on random Voronoi tessellations, with a single free parameter 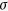 expressing the shape disorder. Although the physical interpretations remain open, this advocates the parameters 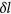 and 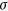 as two independent indicators of the environment in the polar regions, which are easily accessible by remote sensing.

Sea ice is an important constituent of our planet’s surface, covering almost 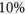 of the oceans. Ice conditions are entangled with environmental changes and can be used to monitor them. In turn, the behavior of ice has consequences on climate, wildlife, and people, deeply affecting many of the processes that take place in the polar regions. Perhaps the most dramatic transformation undergone by polar ice is fragmentation. Cracks are formed on a vast set of length scales, in salty as well as in fresh-water ice. The phenomenology ranges from the long “travelling” cracks in pack ice caused by winds^1^, to the fractures forming in the ice shelves due to glaciological stress fields^2^, down to the breaking of small floes due to collisions.

The measurement of morphological properties of ice floes could inform us on key properties regarding the rheology of sea ice and help the reconstruction of the number of floes of a given scale starting from incomplete measurements or measurements at different scales (e.g., low-resolution satellite data). The rich spectrum of behaviors of sea ice and the available data have already attracted statistical mechanics investigators^3^,^4^,^5^ A general classification of fracture patterns, especially for geological applications, has been proposed very recently^6^. Since many physical phenomena converge to fragment polar ice, a unified view of the process is not available, and not simple to produce. In this situation, the main questions are related to how to interpret the available data and what to extract from them. Ideally, one wants to extract from the complex satellite data simple but highly informative measures of sea-ice morphology. To this end, floe size is an easily-accessible observable, which in general has been fruitfully employed in the characterization of many complex systems^7^,^8^,^9^ as well as of simple ensembles of particles^10^,^11^,^12^ Shape is another, possibly independent, source of information, which may reveal geometric details of the underlying physical processes.

Here, we take an empirical approach to the question of defining useful observables regarding sea-ice morphological properties, and analyze data obtained from satellite images of sea ice detached from the shoreline, called *drift ice*, focusing on the size and shape of the individual floes that drift ice is composed of. Remote sensing makes the access to these data simple, by the exploitation of popular services such as Google Maps. Since floe diameters vary across several orders of magnitude, depending on season, location, and weather, we ask whether common patterns emerge, and how they may be related to the physics of ice fragmentation.

Regarding ice-floe size, while some of the previous studies found evidence for scale-free distributions^5^,^13^ we find that characteristic lengths are present in our data. The distributions we obtain, from data measured in different locations and conditions, all follow the same *finite-size scaling form*, which only depends on a single length scale. Scaling is a significant concept, commonly found in critical phenomena and self-organized criticality, but also in diverse fields including ecology^14^, biology^9^, and the theory of fracture^15^. One notable consequence of a scaling distribution is the possibility of characterizing the whole range of variability through the specification of a single parameter. We rationalize this observed empirical regularity by the use of simple stochastic models of fragmentation. Previous attempts, aimed at explaining full scale invariance^16^, concentrated on hierarchical fragmentation involving the stabilization of fragments^17^,^18^ random multiplicative cascades^13^, or the competition between fracture and healing^5^. We discuss a complementary approach based on a linear model whereby breaking is driven by external sources, whose action is supposed to be characterized by a single length scale, and assumed to be driven by a flat spectrum of perturbations. Importantly, we point out that all available models of crack propagation in brittle solids yield the same results in terms of scaling, and thus, while the single length scale we uncover is an effective measure of the state of fragmented ice, its physical interpretation is elusive and needs to be probed by direct experiments.

These results lead to the conclusion that floe size alone cannot be a fully informative measurement of sea-ice properties. For this reason, we widen the analysis of floe contours by considering also their shapes. Surprisingly, while particle size is widely considered in the literature—e.g., for classifying new fragmentation physics^19^—fragment shape is usually neglected. The distribution of anisotropies has been considered in different settings—such as the explosion of shells^20^ and road networks^21^—but apparently never for ice floes. We propose a simple measure of floe elongation, based on concepts from polymer physics, which measures the anisotropy of the individual floes from the eccentricity of the inertia tensor of their contours. Importantly, such a measure can in principle distinguish between isotropic and non-isotropic physical processes (e.g., lateral melting versus stress failure^22^). Another important feature of elongation is that it is independent of size, as we demonstrate directly with empirical data of sea ice. The distribution of floe elongations measured from satellite data leads us to propose a one-parameter model, describing the shapes of ice floes as the Voronoi cells of randomly-placed seeds. This simple geometric model predicts the observed distributions to a remarkable accuracy, and permits the identification of a dimensionless parameter 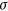, describing the correlations between the positions of the ice seeds. Physically, a possible (but likely simplistic) interpretation for such a model is the process in which freezing water around seed floes makes them enlarge and coalesce during the winter.

In brief, our results can be summarized as follows. (Ia) Ice-floe sizes follow a simple scaling form, hence the size distribution is characterized by a single length scale. (Ib) This feature is predicted by diverse models; therefore, size alone is only moderately informative about the physical processes at play. (II) Ice-floe shape asymmetries are reproduced by a novel geometric model, which should set a more stringent constraint on the possible relevant physical processes. (III) Points (Ia) and (II) suggest two independent scalar quantities—a *characteristic length* and a *shape disorder* parameter—that we advocate as useful for comparing ice-floe images taken in different regions and conditions.

## Floe contours from remote sensing

**Figure 1:**
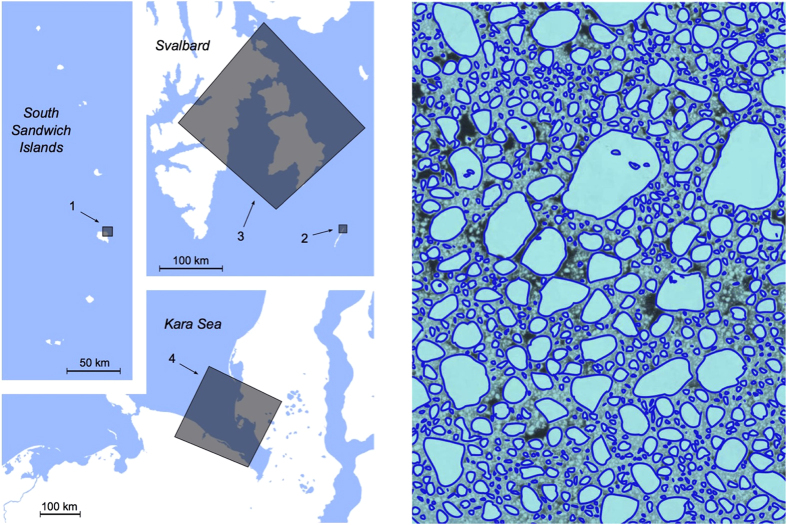
Data were produced by segmentation of satellite images from four locations, indicated by shaded areas in the left panel (1–Montagu, 2–Hopen, 3–Svalbard, 4–Kara). An edge detection algorithm yields the silhouette of each ice floe, provided it is larger than a few pixels. The right panel shows a portion (approximately 15 km wide) of a larger snapshot in data set 3 (light blue, original colors from red, green, and blue bands), merged with the detected contours (dark blue). (The map was drawn with matplotlib^53^.)

We employed four data sets, composed of visible-light imagery taken by two different satellites in four different locations in the north and south hemispheres (see Fig. 1):

1. Montagu Island area (Weddel Sea, south hemisphere); ice floes 2 m to 100 m wide; image taken in October 2003 by the QuickBird-2 satellite; resolution ~2.5 m/pixel; retrieved from Google Maps^23^.
2. Hopen Island area (Barents Sea, north hemisphere); ice floes 2 m to 150 m wide; image taken in June 2009 by the GeoEye-1 satellite; resolution ~1.7 m/pixel; retrieved from Google Earth.
3. Svalbard area (Arctic Ocean, north hemisphere); ice floes 60 m to 5 km wide; image taken in June 2001 by the Landsat 7 satellite; resolution 30 m/pixel; retrieved from the U.S. Geological Survey^24^.
4. Kara Sea (north hemisphere); ice floes 150 m to 5 km wide; image taken in March 2000 by the Landsat 7 satellite; resolution 30 m/pixel; retrieved from the U.S. Geological Survey^24^.

All images were segmented by the Potrace algorithm, and contour information for each detectable floe was gathered. More details about the satellite imagery and the data analysis can be found in the Methods section. We obtained approximately 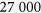, 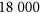, 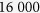, and 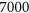 contours for the four images respectively. This data set is limited, but attempts to capture different locations and different seasons, primarily in the marginal ice zones. We regard it as a test case for our observations and methods. Larger sets would be needed for developing more realistic physical models.

First, we focus on the linear size of the floes. The segmentation procedure yields each contour 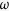 as a closed polyline, identified by a set of 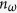 nodes 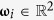 with 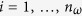. To extract a linear measurement of floe size, we consider the square root of the mean square distance of all contour points from the center of mass (an analog of the “radius of gyration” used in polymer physics). We weigh each point by its distance from the preceding one, in order to compensate for the nodes being spaced unevenly along the contour. This defines a floe “size” 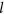 as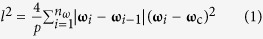 where 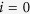 is equivalent to 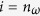 by convention, 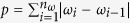 is the perimeter of the contour, and 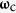 is the center of mass
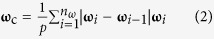
Note that measuring the linear size via the perimeter as 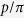, which is more sensitive to the roughness of the contours, does not affect heavily the results presented hereafter.

**Figure 2:**
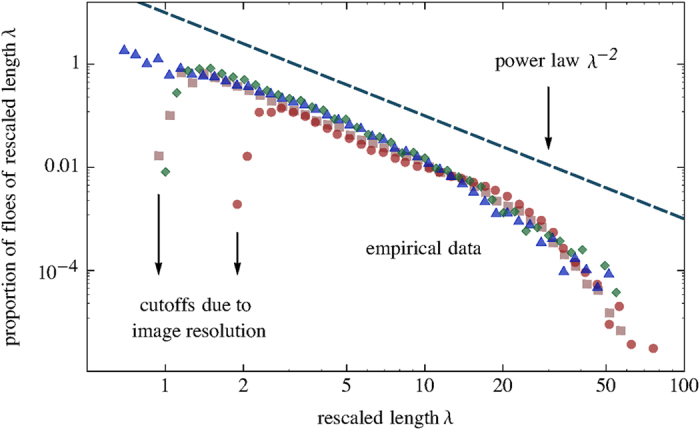
Size distributions of floes obtained from satellite images in four dissimilar conditions collapse well on the same curve. The rescalings on the x-axis were adjusted by eye from the cutoff points, and the normalization constants corrected accordingly. Circles, squares, triangles, and rhombi correspond to data sets 1, 2, 3, and 4, respectively. The dashed line shows a power law of exponent 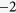 as a reference. The drop-off at small sizes is due to the finite resolution of the images; data in this region are discarded in Fig. 3.

The measurement of 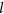 for all floes in the four data sets gives the four corresponding size density distributions 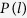. Inspection of the curves indicates that a power-law regime is present in all data sets (with exponent 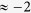), followed by a smooth cutoff for large floes. We found that these curves collapse rather well onto a single curve by a simple rescaling of the two axes (Fig. 2). This suggests that each curve is characterized by a single length scale. The small-size drop-offs visible in the plot are due to the underestimation of floes of small size, close to the resolution of the images, and are therefore not universal. Unfortunately, this purely technical feature makes it impossible to follow an accurate approach to data collapse, in the spirit of^25^; the rescaling has to be eyeballed. However, albeit of an empirical nature, the collapse is remarkable, considering that the data come from diverse locations and seasons, and involve broadly dissimilar scales.

## Fragmentation theory for the size distribution

To rationalize the observed scaling behavior, we consider statistical models of fragmentation. We briefly review two classes of existing models; the first is a stochastic dynamics for the number of floes of a given size, the second is a deterministic geometric description of crack propagation in brittle fracture^26^,^27^. Then we introduce a third alternative model, and argue that the emergence of a single characteric length scale is compatible with all these scenarios.

In the first class of models, the physical aspects of the fragmentation process are summarized by a rate 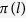 at which a floe of size 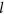 breaks down into two smaller objects. At a mean-field level description, each floe is supposed to experience the average environment, i.e. spatial fluctuations are neglected. We consider a system in which the fraction of floes having linear size *l* at time 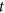 is 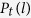. The distribution 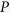 obeys the following rate equation^28^:
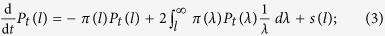


the first term is due to floes of size 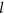 breaking down into smaller floes, the second term is due to larger floes of size 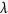 generating fragments of size 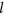 (in the 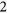 possible ways), the factor 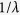 has geometric origin (in its absence, the in-flow from larger floes would be rescaled by a factor proportional to their size); the last term 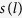 is a generic source, which may represent for instance the generation of large floes by the fracture of ice fields.

When no source is present, 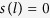, the fragmentation dynamics (3) is known^29^ to produce a scaling size distribution 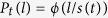, where 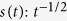 is the decreasing typical floe size. If the breaking rate scales as the area of the floe, 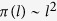, then the scaling function, at large 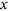, is 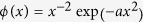, which reproduces the exponent 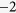 exposed in Fig. 2, and also predicts a universal exponential cutoff. An interpretation based on such a dynamical picture links the characteristic scale of floes to the time passed since the beginning of the fragmentation process, that is of the melting season. The data considered here were not sufficient to test these correlations and further investigations are needed.

The second class of available models is that of brittle fracture. Fragment size distributions are obtained by considering the propagation of cracks in the material. Older theories^30^ considered smooth cracks, while more recently it has been recognized that beyond a critical speed they can become unstable and branch^31^. Moreover branches attract each other and therefore merging of cracks has been included in the picture. A well-established theory^32^,^33^ yields a fragment size distribution dominated by a hierarchical process, whereby a number of cracks stem from a main fissure and then merge, the longer-lived cracks giving rise to the larger fragments. In this description, again, the distribution of the linear floe sizes 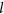 is a power law of exponent 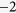 cut off by an exponential in 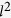. The upper cutoff represents the single length scale of the system.

Additionally, we propose here a complementary formulation belonging to the first kind of models, which assumes that the empirical distributions could be the *stationary* states of processes happening at time scales much faster than the seasonal variations. The advantage of this description is that it provides a natural setting for the observation that the same scaling form agrees with empirical data taken in different periods of the year (compare e.g. data from the Svalbard area, taken in June, with those from the Kara Sea, taken in March).The steady state 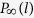 is obtained by setting the time derivative of 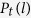 to zero in Eq. (3). Note that technically the presence of a source 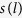 is necessary in order to have a steady state at all, since its absence would require an unbounded distribution 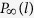. Setting 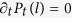 in (3) and taking the derivative with respect to 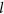 yields an ordinary differential equation whose solution is
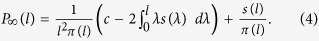
We consider the case where the source generates floes of a fixed size 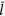 with a rate 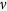, i.e. 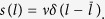 This includes the case of no source (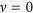). The solution, away from 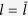, is 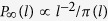: the size dependence of the rate 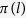 completely determines 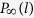.

In order to proceed, one has to specify 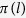. We present here a generic argument for the scaling of 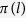, which is suggested by the yearly process of sea-ice refreezing^5^,^18^ whence water interfaces between different floes are rejoined. We make the simplifying assumption that the only relevant scale is that of the coalesced fragments, 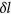. Supposing that a floe of linear size 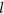, resulting from the coalescence of 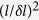 smaller floes, breaks along the junction lines between them, then it can fracture in roughly 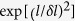 different ways. If these are admitted to be independent then the breaking rate will be 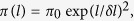 where 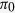 is a constant. The form of the breakage rate resulting from this argument can be used in Eq. (4) to obtain a steady-state solution for the floe-size distribution. In particular, the steady state solution in the absence of source is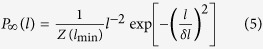 where 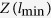 is a normalization constant. The divergence in 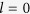 is not integrable, therefore a cutoff 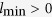 needs to exist. Physically, this cutoff can represent an elementary scale under which fragments cease to divide (this happens for instance in dust aerosols^11^), or under which melting becomes dominant. In the analysis of the satellite images considered here, the lower cutoff is not physical, but corresponds to the resolution of the images (pixel size), as can be seen in Fig. 1, where tiny floes are visible that are not revealed by the algorithm. The model—that is 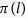, and therefore 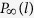
—is then characterized essentially by the single scale 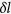 (since the lower cutoff only affects the normalization).

It should be stressed that all models are necessarily simplified descriptions and neglect some of the phenomena caused by the very complex interaction between sea ice and the environment^34^. A most important one is melting due to the seasonal temperature variations. Lateral melting^35^ would be represented by a term 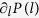 in (3), thus possibly breaking the scaling form of the stationary state. The regularity observed can be then interpreted as a measure of the marginality of lateral ice melting as opposed to fracturing, in the regimes considered. Note that melting is supposed to become more relevant at smaller scales; our data suggest that higher-resolution images are necessary to this aim. Indeed, aerial photography suggests that a different behavior sets in at small sizes^36^,^37^ Moreover, the source term could introduce deviations from the source-free steady state, as expressed by (4), which could be detected by studying the size distribution in the marginal ice zone close to the fracturing pack ice^22^. Additionally, these “mean-field” descriptions disregard floe shape, as well as the isotropy of fissures^20^,^38^.

**Figure 3:**
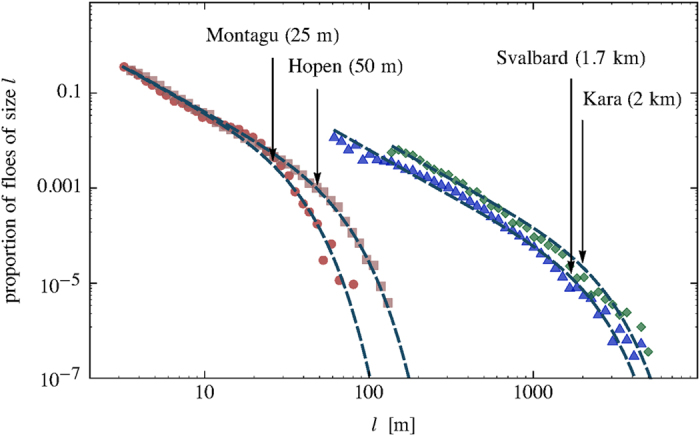
Empirical floe sizes in the four data sets are well described by a statistical model of fragmentation, characterized by a single crossover scale (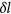 in the text), indicated by the arrows. The dashed lines are the steady states (5) for the four corresponding characteristic lengths; the lower cutoffs 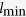 on the distributions have been fixed to the positions of the maxima in Fig. 2, and data below those thresholds have been discarded.

Summing up, the three models described above are compatible with the following scaling form for the fragment size distribution,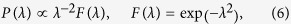 where the scaling variable is 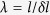. This expression is in very satisfactory agreement with the empirical data. Fig. 3 compares the theoretical prediction (5) with the empirical data from our four data sets. Some previous studies claimed power-law regimes with exponents possibly deviating from 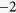
36,39 However, there is some debate around the numerical solidity, the interpretation, and the practical significance of this variability^37^. It is possible that some of the deviations from the scaling form (6) found in other studies came from the superposition of data having two or more length scales.

## Characterization of floe shapes

The previous section has shown that the characteristic length 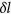 may be subject to very different interpretations, and even the question on whether the scenario is dynamic or stationary seems left open. In this section we propose an additional observable, based on the shape of the floes, which is, as we will show, independent of their size.

When considering contour data, the gyration radius used in the definition of 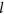 in Eq. (1) is only one of several scalar quantities that can be constructed starting from a tensorial object, called gyration tensor. The gyration tensor, in the simple case of a set of point particles with unit mass, is exactly the inertia tensor, describing the rotational degrees of freedom of the system (the points are supposed to have fixed relative positions). It provides a compact description of some properties related to the shape of contours and lattice walks, and is fruitfully employed for instance in polymer physics^40^,^41^,^42^,^43^ Here we use a slightly modified version, which takes into account the different “masses” corresponding to the different step lengths between the contour points 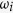 (see also the definition of 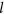 above). In a fixed coordinate system 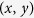, the gyration tensor is a symmetric matrix 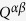, where 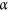 and 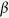 can take values corresponding to the two coordinates 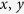, defined as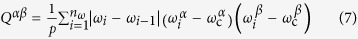 the center of mass 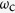 is defined as in (2), and 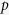 is the perimeter. In this form, 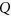 is (an approximation of) the inertia tensor of a uniform mass distribution lying on the perimeter of the floe, and is therefore a proxy for symmetry properties related to its shape. The symmetric matrix 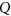 has two real eigenvalues, 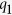 and 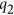, representing the moments of inertia with respect to the two principal axes of rotation. Notice that the square of the gyration radius is 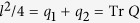. A functionally independent object is the *shape factor*,
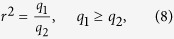
which expresses the elongation of the object. Specifically, it is the inverse of the ratio between the area of the inertia ellipsoid of the floe (which approximates its surface) and that of the smallest circle that contains it. For a spherically symmetrical floe (and for all regular polygons) it takes its minimal value 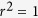, while 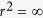 for a segment. Its square root 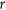 is the *aspect ratio*, i.e., the ratio between the lengths of the longer and the shorter axis.

**Figure 4:**
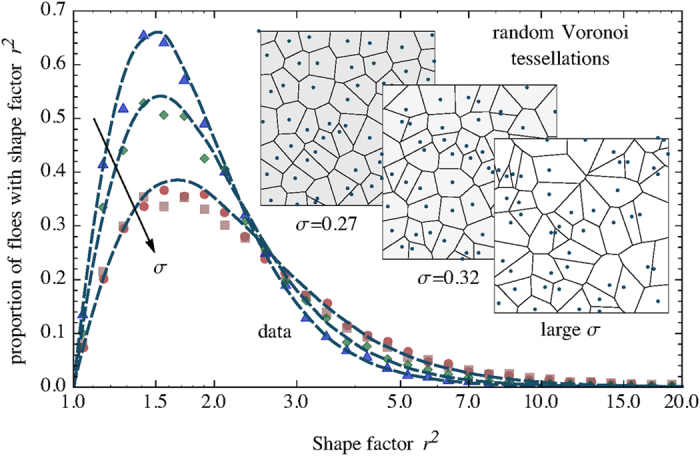
The shape factors measuring the elongation of the floes are reproduced by random Voronoi tessellations. The plots show the shape factor distributions for data and for Voronoi tessellations where the underlying point process (describing the locations of the freezing seeds) has disorder parameter 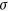. Symbols are the empirical data (Svalbard 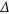, Kara 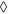, Montagu 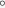, Hopen 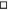), dashed lines are obtained by simulations of the random Voronoi model. The insets show the snapshots of three realizations, corresponding to the best-fitting values of 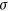.

We have measured 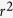 for all contours in the four data sets. The empirical distribution functions (shown in Fig. 4) are peaked around 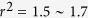, with a fast rise after 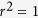 and a slow drop-off at large elongations. These histograms are smooth and unimodal, suggesting the existence of a single dominant mechanism underlying their emergence.

To interpret and characterize these data, we introduce a simple stochastic geometric model, whose only parameter is linked to the distribution of seed locations. Specifically, the seeds are placed randomly following what is known as a “simple point process” in probability; the actual implementation is described below. To fix the ideas, this model may be interpreted as describing the refreezing of floes during winter as the coalescence of radially growing ice floes started from randomly-located seeds, but its formulation is purely geometric and one cannot exclude that it may be derived from alternative physical interpretations. Once the seeds are fixed, the shapes of the resulting floes are obtained as the cells of the *Voronoi tessellation* generated by the seeds^44^. A Voronoi tessellation is a partitioning of the plane into regions (cells), based on the distances from a set of special points (seeds). Each point in the plane (minus a set of zero measure) is associated to its closest seed. A cell is the set of all points associated with a given seed. In other words, floes are the domains of proximity of the freezing seeds.

**Figure 5:**
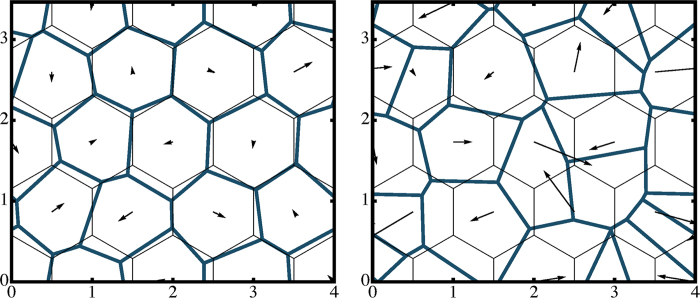
Illustration of the random tessellation model, which describes the shapes of ice floes as the Voronoi cells of freezing seeds. Seeds’ locations (here with 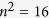 seeds) are obtained by perturbing a regular triangular lattice by Gaussian random displacements with variance 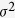 (arrows in the drawing, 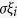 in the text); 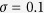 in the left panel, 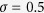 in the right panel. The thin lines are the Voronoi cells of the triangular lattice, the thick lines are those of the seeds, i.e., the shapes of the floes. The rectangular region depicted is wrapped around a torus in order to eliminate boundary effects.

The simplest point process is arguably the Poisson point process, whose realizations are sets of random independent points. We employ the following generalization, aimed at introducing repulsive correlation between points in an easily controllable way. Refer to Fig. 5. Fix an integer 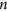 (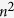 will be the number of seeds) and consider a rectangular region 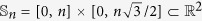, with periodic boundary conditions. Let 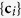, *i*=1, …, *n*^*2*^, be the points of a regular triangular lattice embedded in 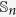, with lattice spacing 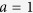. It realizes a regular triangulation of the torus. The seed positions 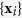, 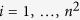, are random variables defined as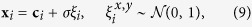 where the noise terms 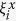 and 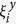 are independent identically-distributed Gaussian random variables with zero mean and unit variance. The quantity 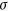 then expresses the departure from perfect order. The perfect lattice is realized for 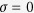, in which case the Voronoi cells are all regular hexagons with shape factor 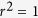; the opposite limit 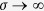 recovers the Poisson point process.

**Figure 6:**
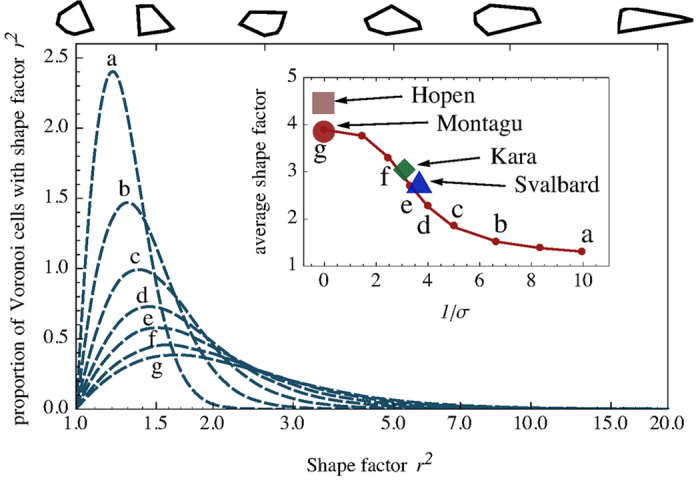
A one-parameter random Voronoi model interpolates between perfect order (a delta-shaped distribution centered in 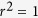) and Poisson-Voronoi random tessellation. Curves “a” to “f” are obtained by the model with increasing values of the shape disorder 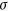; the curve “g” is obtained by the Poisson-Voronoi model. The inset shows how the average shape factor (corresponding to the curves in the main panel and to empirical data) is related to the inverse of the shape disorder 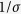 (red line).

Simulating the model for several values of 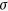 allowed us to choose, for each data set, the value of 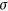 that best fitted the empirical distribution of shape factors. Fig. 6 displays the theoretical distributions obtained by simulation, and shows that, as expected, 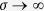 converges to the random Poisson-Voronoi case, while a delta-shaped distribution centered at 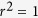 is approached for 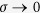. The agreement between this geometric model and the empirical data is impressive (Fig. 4 and inset of Fig. 6), suggesting that the phenomenology encoded in the elongation of ice-floe shapes can be completely characterized by a single parameter, measured by the average shape factor, or, equivalently, by 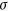. We therefore propose 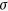 as a measure of the “characteristic disorder” in the distribution of the ice floe seeds.

**Figure 7:**
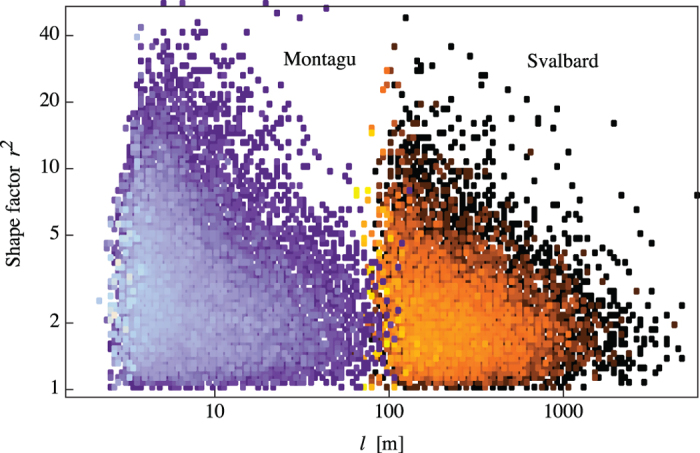
Elongation and size of floes are uncorrelated. The scatterplots are obtained from empirical contours in the Montagu (blue cluster on the left) and Svalbard (orange cluster on the right) data sets. Lighter colors correspond to higher density of points.

The solidity of such a measure is supported by the following observations. (i) Results are not sensitive to the number of seeds (in the simulations reported in the figures, we sampled 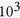 realizations with 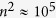 points for each 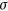, but the results are indistinguishable from those at 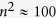). (ii) Choosing a specific lattice (for instance square or hexagonal) and probability distribution of 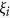 does not affect the agreement with the empirical data, apart from slight readjustments of the fitted values of 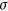. (iii) Surprisingly, and importantly, the shape factor of a single floe is uncorrelated to its size, as shown by the scatterplots in Fig. 7 for two data sets with very different characteristic lengths (the other two are similar). This last observation indicates that the shape properties are decoupled from the size distribution, and justifies the description of the floe shaping mechanism as a Voronoi tessellation for a wide range of length scales.

## Discussion and Conclusions

The sea ice system is complex, and several processes are at play, influencing each other. Further data analysis and modeling may attempt to assess, for instance, the relevance of melt pond formation on the thinning surface, which enhances fracturing, or of the development of pressure ridges. Our data are relatively limited, and leave the physical interpretations open, but the regularities observed in the floe shapes express important constraints that any realistic theory should satisfy. The main result of this work is the introduction of two novel independent scalar observables, or “order parameters” for ice floes, summarizing the properties of the distribution of sizes and of shape anisotropies respectively. Regarding floe size, data and different models of fracture suggest a scenario where the spectrum of the different forces at play is reducible, to a good approximation, to a single dominant scale, 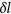. This parameter contains all the information concerning the complex environment, including the ocean, the winds, and the ice floes themselves, relevant to construct the length distribution. Both geometric and mean-field models of fracture and fragmentation can account for the empirical size distribution 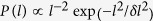. This shows that independent measurements linking floe size to environmental history, conditions, and perturbations are needed to gain most physical insight. Different models assign different interpretations to the length scale 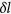, which is the only distinguishing feature of our four data sets as long as floe size is concerned. Note that the assumption of stationarity corresponds to considering long times; this is realized in practice if the dynamics of fracture is faster than the seasonal variation of parameters. The scale 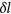 is probably affected by the annual changes in temperature and by other environmental conditions, and it seems reasonable to expect it to vary on time scales longer than the one driving equation (3). A systematic study of the variations of 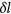 in a given location could give important clues on the changing climate and environment. Possibly, the characteristic length could provide an indicator of ice thickness^18^, which is an important parameter in the interactions between ice and ocean waves^22^. While several fracture theory and fragmentation scenarios may account for the fact that floe sizes have ubiquitous characteristics, we cannot exclude that other physical processes are relevant to establish this. For example, the wave field on the surface of the ocean, might be determinant for setting the single length scale controlling the floe size distribution. Additionally, the emergent characteristic length is likely affected by multiple important factors such as season (growth *vs* melting), location (marginal *vs* consolidated) and ice age and type (saline recently formed *vs* fresher older ice).

Considering shape elongation, we find that, as in the case of floe size, a single parameter fully characterizes the distribution in the whole available range of variability. However, differently from size, we found no universal scaling function accounting for the shape anisotropy data. On the other hand, we showed how a simple geometric model of ice accretion and coalescence (related to random Voronoi tessellations) realizes a one-parameter family of distributions fully capturing the shape fluctuations of ice floes, condensing once again the statistics of the elongations to a single dimensionless parameter, the shape disorder 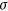. The remarkable agreement between model and data validates the interpretation of 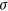 as a physically relevant observable. The precise identification of the physical processes responsible for shaping the observed distributions is a difficult task, and remains an open question. Floe elongation distributions may be sensitive to the specific conditions mentioned above (season, location, ice age and type), and future systematic data-analysis studies may be able to assess these features.

It would be interesting to study the shape of ice fragments in other systems, both geological (e.g., the CO^2^ ice layer on Mars^45^) and in the laboratory^16^. The parameter 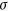 is possibly related to the packing properties of ice just before the freezing season^3^. In line with the stationary model for floe size, the random Voronoi tessellation model suggests that shape anisotropies might be the product of fracturing mechanisms facilitating separation along the junctions between coalesced floes. Plausibly, more regular lattices in the model (lower values of 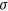) correspond to closer packing, and thus to stronger interaction between the floes. Curiously, random Voronoi tessellations are used as a phenomenological protocol for generating realistically-looking fracture patterns in computer graphics^46^.

These results could have implications for the rheology of sea ice (a description of ice in the marginal ice zone as a non-Newtonian fluid has been attempted by some authors^47^). A potential practical application of such results could be the reconstruction of the number of floes of a given scale starting from incomplete measurements or measurements at different scales. For instance, consider the situation where one has access to satellite data at low resolution (large 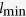), which include only large floes, beyond the crossover scale. Then the full curve can be reconstructed, by fits against the scaling form, and the number of smaller floes can be indirectly evaluated.

## Data and methods

### Data sets

We give here more details about the satellite imagery used. Each data set is composed of a single satellite image. Image 1 (Montagu Island) was taken by the QuickBird 2 satellite on October 16, 2003; catalog ID: 1010010002631B00^48^; we used the top right quadrant of the image covering an area of 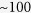 km^2^, available in high resolution through Google Maps. Image 2 (Hopen Island) was taken by the GeoEye 1 satellite on June 13, 2009; catalog ID: 1050410001E37000^48^; We used a small portion of around 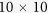 km^2^ in the north-east sector of the image, available in high resolution through the Google Earth application. Spectral bands and exact resolutions for images 1 and 2 could not be determined; if one assumes, as is likely, that Google services use multispectral (red+green+blue) images then the pixel resolutions are 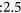 m for image 1^49^ and 1.7 m for image 2^50^. Images constituting sets 3 (Svalbard; taken on June 5, 2001; catalog ID: LE72100042001156AGS00^24^) and 4 (Kara Sea; taken on March 17, 2000; catalog ID: LE71670112000077SGS02^24^) were taken by the Landsat 7 satellite, with the “Enhanced Thematic Mapper Plus” instrument; bands 1,2, and 3, corresponding to wavelengths in the red, green, and blue visible light respectively, were merged; the pixel resolution is 30 m. Both images have an approximate spatial extent of 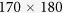 km^2^. Only the quadrants with less than 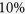 cloud coverage were selected.

### Segmentation method

All images were first made monochromatic by application of a threshold on pixel intensity. This threshold is gauged by sight, but we tested that its precise value does not have a relevant impact on the results, as long as it lies between the average ice value and the average background value. Then the contours of single ice floes were extracted (Fig. 1) using the Potrace algorithm^51^. The main steps of the algorithm are as follows. (I) The bitmap is decomposed into paths, separating black and white regions. Square lattices (as the pixels in an image) present an ambiguity in the definition of clusters, when two contours meet perpendicularly at a vertex; we used the prescription of connecting preferentially black components in these cases. (II) After despeckling (discarding paths enclosing only 1 or 2 pixels), paths on the square lattice are converted into polygons, following a parameter-free optimization phase (details are in the documentation^51^). The full algorithm includes a further step—based on aesthetic principles—aimed at detecting sharp corners and smoothing out the others, which we skipped.

### Remarks on the cutoffs

Figure 1 shows a non-negligible area of unsegmented ice, mainly due to two components. (I) Very small floes, below the despeckling threshold; this is due to the pixel resolution, as discussed above, and is exposed by the quick drop-offs of the curves in Fig. 3. (II) Darker regions, below the intensity threshold; this is a more serious limitation, as it reduces systematically the estimate of the distributions at low sizes. It is probably responsible for the slight deviations from power law that are visible in some of the curves in Fig. 3.

While pixel size imposes a cutoff on the minimum floe-size detectable, image size has an influence on the statistics of large objects. Underestimation of the number of large floes can be quantified^52^; for instance, a circular floe of 5 km in diameter at least partially covered by a Landsat 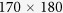 km^2^ snapshot has a 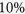 probability of being segmented incompletely. Notice that such a small change would be undetectable in the logarithmic plots in Fig. 2 and Fig. 3. We manually removed all partially covered floes from the data sets.

## Additional Information

**How to cite this article**: Gherardi, M. and Lagomarsino, M. C. Characterizing the size and shape of sea ice floes. *Sci. Rep*. **5**, 10226; doi: 10.1038/srep10226 (2015).
